# Clustering Gene Expression Regulators: New Approach to Disease Subtyping

**DOI:** 10.1371/journal.pone.0084955

**Published:** 2014-01-09

**Authors:** Mikhail Pyatnitskiy, Ilya Mazo, Maria Shkrob, Elena Schwartz, Ekaterina Kotelnikova

**Affiliations:** 1 Institute of Biomedical Chemistry, RAMS, Moscow, Russia; 2 Ariadne Diagnostics LLC, Rockville, Maryland, United States of America; 3 Elsevier Inc, Rockville, Maryland, United States of America; 4 Institute for Information Transmission Problems, RAS, Moscow, Russia; University of Miami School of Medicine, United States of America

## Abstract

One of the main challenges in modern medicine is to stratify different patient groups in terms of underlying disease molecular mechanisms as to develop more personalized approach to therapy. Here we propose novel method for disease subtyping based on analysis of activated expression regulators on a sample-by-sample basis. Our approach relies on Sub-Network Enrichment Analysis algorithm (SNEA) which identifies gene subnetworks with significant concordant changes in expression between two conditions. Subnetwork consists of central regulator and downstream genes connected by relations extracted from global literature-extracted regulation database. Regulators found in each patient separately are clustered together and assigned activity scores which are used for final patients grouping. We show that our approach performs well compared to other related methods and at the same time provides researchers with complementary level of understanding of pathway-level biology behind a disease by identification of significant expression regulators. We have observed the reasonable grouping of neuromuscular disorders (triggered by structural damage vs triggered by unknown mechanisms), that was not revealed using standard expression profile clustering. For another experiment we were able to suggest the clusters of regulators, responsible for colorectal carcinoma vs adenoma discrimination and identify frequently genetically changed regulators that could be of specific importance for the individual characteristics of cancer development. Proposed approach can be regarded as biologically meaningful feature selection, reducing tens of thousands of genes down to dozens of clusters of regulators. Obtained clusters of regulators make possible to generate valuable biological hypotheses about molecular mechanisms related to a clinical outcome for individual patient.

## Introduction

Patient stratification or personalized approach to therapy is one of the most perspective fields in the modern medicine. Finding different biological patterns within the group of patients with the same diagnosis could lead to more precise and effective prescriptions. To address this issue it is necessary to reveal different mechanisms within the same disease, find novel biomarkers and develop new diagnostic tests that would accurately classify patients into homogeneous diagnostic or prognostic subgroups. Gene expression studies stimulated the great progress in this field.

In the past decade numerous papers were published claiming successful application of gene expression analysis to patients subtyping and prediction of survival. A typical study includes the application of statistical techniques based on supervised learning or cluster analysis to group samples based on their expression profiles.

However, an observation made by many researchers is that there is little overlap in gene signatures and lists of potential biomarkers between studies [1], [2], [3]. For example Michiels *et al* [2] reanalyzed seven studies that have attempted to predict prognosis of cancer patients based on expression profiles and reported that lists of predictor genes were highly unstable and strongly depended on the selection of samples in the training sets. Gene signatures constructed in three separate studies of colorectal cancer shared only one common gene [4]. Venet *et al* [5] has shown that in breast cancer any set of more than 100 randomly selected genes has a 90% chance to be significantly associated with outcome.

There are many reasons for observed lack of overlap between signatures. Technical factors include usage of different platforms for analysis of gene expression and different normalization methods. Statistical analysis is complicated by the fact that in typical expression study number of features greatly exceeds number of samples (“curse of dimensionality”) which often leads to overfitting and poor performance of feature selection methods. Although a lot of work has been done in this area (for reviews see [6], [7]), problem of selecting variables in high dimensional classification is an ongoing research.

Several biological factors also contribute to discrepancy between lists of prognostic genes. One of them is intra- and inter-individual variance in clinical studies [8]. Another factor is high level of expression correlation between genes which cooperate together to execute their function. Since the strength of correlation varies between training sets this results in unstable rank order of discriminating genes in the prognostic signatures [9].

Finally standard statistical methods for patients' classification ignore existing well-established biological relationships between genes. This limits interpretation of generated signatures and results in poor progress for the translation of gene expression signatures in clinical practice [10].

One possible way to address these problems is to interpret the expression data at the level of functional groups of genes such as signaling and metabolic pathways. Genes are mapped onto predefined gene sets (usually taken from KEGG pathways or Gene Ontology categories) and activity scores of gene sets describe patient profile. Matrix of gene set activities is further used in cluster analysis or supervised learning to perform disease subtyping. For example in PathOlogist method [11] expression data are normalized in a special way [12] and further used to characterize set of pathways with activity and consistency scores. The set of scores for each pathway allows performing several types of analysis including binary classification (e.g. cancer vs normal), correlation (e.g. response to treatment) and survival prediction. Molecular analysis at the pathway level gives more reproducible results and there is much more overlap between studies at the level of pathways [13], [14]. Another benefit is biological interpretability: genes in gene sets are already grouped according to their cellular role.

In one of early studies [15] Breslin *et al* utilized signal transduction pathways from TRANSPATH/TRANSFAC databases to assess signaling pathway activity. Pathway activity was calculated as a sum of normalized expression values for all downstream target genes of the pathway. Although Breslin *et al* did not use supervised or unsupervised learning techniques they've shown association between sample-wise pathway activity and clinical classification, thus confirming relevance of pathways for understanding biology of disease subtypes.

Two types of approaches have been proposed to describe the activity of a given pathway based on expression of the constituent genes. First group of methods does not require sample class assignment and use unsupervised measures such as mean or median of all genes within a set [16], [17] or first principal component of a gene expression profile [18]. However, the main limitation of these methods is that some genes from a pathway may have expression which is not correlated with phenotype of interest. Such genes will increase overall noise in the data and reduce classification accuracy. To address this problem second group of methods utilize supervised approaches such as naïve Bayes model [14] and condition-responsive genes (CORGs) [13]. However CORG-method is not sensitive to small but coordinated changes in expression and Bayes approach relies on accurate estimation of probability density function for each class requiring relatively large sample size. While the supervised approach seems to be more accurate, the between-samples dependencies complicate application of these methods on a single-patient basis.

To facilitate biological interpretation of observed changes in expression gene set enrichment methods have been introduced. Widely used gene set enrichment analysis (GSEA) [19] determines which of a priori defined sets of genes exhibit significant co-operative changes in expression between two conditions. There are several attempts to modify gene set enrichment methods in order to apply them for disease subtyping [13], [20]. For example authors of recently introduced GSVA, Gene Set Variation Analysis [21] calculate sample-wise rank-based enrichment scores, thus transforming coordinate system for expression data from genes to gene sets. Authors demonstrate usage of GSVA score matrix for differential pathway activity identification and survival prediction.

One way to extend gene set enrichment methods is to take into account information about pathway topology. One of the examples of such methods is SPIA [22] where for each KEGG pathway two types of evidences are combined: over-representation of differentially expressed genes and pathway perturbation measured as propagation of changes in gene expression through the graph topology. In PWEA method [23] topological influence factor for each gene is calculated to weight the Kolmogorov-Smirnov statistic used in enrichment analysis. Another approach to extension of GSEA with network context was proposed by Alexeyenko *et al* [24]. For their method, Network Enrichment Analysis, authors compiled global network with more than 1,400,000 functional links. Using this background network algorithm identifies functional gene sets with significant number of links connecting this gene set with differentially expressed genes in each patient.

One disadvantage of enrichment methods is their dependence from *a priori* defined gene sets. Most of methods use sets of functionally related genes derived from Kyoto Encyclopedia of Genes and Genomes (KEGG) [25], Gene Ontology (GO) [26] or MSigDB [27]. However this approach cannot identify novel interesting gene sets (e.g. activated signaling and regulatory cascades) which may give clues about individual aspects of disease development. Predefined gene sets also seem biased, for example KEGG database is more oriented to metabolic pathways, while accurate GO enrichment is complicated by entangled structure of ontology.

Some methods were developed which explore the idea of finding upstream network regulators using expression data. For example Kel *et al* [28] suggested analysis of promoters of differentially expressed genes in order to find transcription factors responsible for observed changes in expression. Separate tool identifies upstream signaling molecules (master regulators) which activate/inhibit found transcription factors thus providing causal interpretation of gene expression and ‘reverse engineering’ the signal transduction network involved in disease development. However Kel's approach includes definition of differentially expressed genes and thus may miss small but coordinated changes in gene expression which can be found by enrichment-based methods.

Here we propose an approach to disease subtyping which heavily relates on Sub-Network Enrichment Analysis algorithm (SNEA) [29]. SNEA, an extension of GSEA, given transcriptomics data identifies gene sets with significant concordant changes in expression between two conditions, for example disease and control samples. While most GSEA-based methods utilize predefined gene sets, distinctive feature of SNEA is the construction of gene sets “on the fly” using global network of protein regulation. Each gene set (subnetwork) consists of central entity (“seed”) and downstream genes known to be affected at the expression level by the “seed”. The biological idea behind SNEA is that if the downstream expression targets of the “seed” contain more differentially expressed genes than expected by chance, then the “seed” is one of the activated regulators of the differential expression profile. Regulator often is a transcription factor, but also can be another type of entity which does not even need to be measured in experiment – complex, functional class, small molecule. Information about regulations is automatically derived from scientific literature using text-mining tool MedScan [30], [31] and stored in a database.

Comparing SNEA and GSEA we note that both approaches are very similar, since SNEA is simply GSEA with genesets constructed from global network of interaction, promoter-binding and other events underlying cellular machinery. One advantage of SNEA comes from limitations of available gene sets. There is no comprehensive and universally acknowledged collection of pathways and it is difficult to unambiguously draw the borders between pathways because of cross-talk phenomenon. In addition it is not clear how the pathways change in a disease. Another advantage of SNEA is related to the approach of preparing genesets (subnetworks in SNEA terminology). Selecting genes known to be downstream of a regulator allows for detecting proteins (e.g transcription factors) whose activation (by phosphorylation or translocation) rather than gene expression per se contributes to the condition. Finally identified subnetworks represent more than a simple gene set, they pinpoint the transcriptional regulators underlying biological mechanisms thus suggesting an explanation to the observed expression data.

We consecutively apply SNEA for each differential expression profile, identifying regulators responsible for driving sets of genes showing co-operative patterns of expression. In order to reduce noise influence and increase biological interpretability we group together expression regulators found in all analyzed samples with similar sets of downregulated genes, forming clusters of regulators. Each cluster of regulators is characterized with an activity value, describing expression of downstream genes. Obtained matrix of cluster of regulators activities is used to perform cluster analysis of patients for disease subtyping.

We demonstrate utility of the proposed approach using two transcriptome studies. Dataset GSE4183 [32] describes gene expression in colon biopsy specimens with precancerous adenoma, colorectal carcinoma and inflammatory bowel diseases. Our method groups together patients with similar clinical subtypes outperforming PAM clustering of gene expression data and GSVA method. We also show an example of biological interpretation of obtained results, suggesting regulators involved in colorectal adenoma-carcinoma sequence. Another expression dataset GSE3307 [33] contains comparative profiling of 12 neuromuscular diseases and we demonstrate how our method can be used to group together diseases with similar patterns of expression regulation rather than patients. Disease clusters are evaluated based on biological expertise and we show that proposed method gives more meaningful results compared with traditional expression-based Ward's clustering.

Overall we propose a novel unsupervised approach for patients subtyping based on activity of significant gene expression regulators. Developed technique can be viewed as a biology-driven feature selection method since from tens of thousands of genes we move to hundreds of regulators and further to dozens of regulator clusters. Analysis of clusters of regulators suggests biological interpretation of molecular mechanisms activated in specific conditions. This also enables rational selection of biomarkers specifically downstream from the identified activated regulators.

## Materials and Methods

### Datasets

Two expression datasets (GSE4183 and GSE3307) were obtained from public repository — NCBI Gene Expression Omnibus database, http://www.ncbi.nlm.nih.gov/geo/. Samples in NCBI GEO are labeled with unique digit identifier without referring to the patient's name, so the data were analyzed anonymously. Datasets GSE4183 [34] and GSE3307 [33] were approved by the ethical committee of the Semmelweis University and IRB ‘Candidate gene and protein studies in neuromuscular disease’ correspondingly.

The main requirement for the selection was that dataset should contain control group and at least two other groups of patients corresponding either to different diseases or disease subtypes. Also each group should have included at least five samples. Intensity values were log-transformed and normalized to zero mean and unit variance. For synonymous probesets corresponding to the same gene, the only probeset with maximum intensity was selected.

### Sub-Network Enrichment Analysis

We used SNEA implementation in Pathway Studio 9.0 from Elsevier [35]. Method identifies subnetworks containing central regulator (including but not limited to transcription factors) and downstream target genes which have significantly co-operatively changed their expression. Algorithm starts with selecting the central "seed" from one of relevant entities (protein, complex, or set of proteins, “functional class”) in the database. Database (called Resnet) stores literature-extracted biomedical entities and their relations. At the moment of our study Resnet contained 112097 proteins, 407 complexes and 2977 functional classes. SNEA creates a subnetwork by retrieving all entities interacting with the selected seed. We used two types of interactions – Expression (300465 relations in Resnet) and PromoterBinding (18153 relations in Resnet). Next algorithm uses Mann-Whitney U-test to calculate the p-value for difference between distribution of expression values of regulator's downstream genes and background distribution of all expression values for the selected sample in the experiment. During distribution calculation, the expression value for each entity connected to a seed is accounted for as many times as the connectivity of that entity in ResNet in order to correct for the bias introduced by hubs. Finally subnetworks are ranked according to p-values and top 100 subnetworks with p-value smaller than 0.05 are returned by default.

We modeled one normal “patient” by averaging each gene across all samples from the control group and calculated corresponding log-ratios for each sample outside the control group. SNEA was run on these log-ratios, resulting in list of top 100 significant regulators for each sample from the disease class.

### Regulators clustering

Similarity between pair of regulators was defined as percentage of common downstream expression targets, which is equivalent to Jaccard distance between two gene sets. We calculated distance matrix for all identified regulators and clustered them using Ward's method. To obtain reasonable number of regulator clusters we used method of maximizing average silhouette [36] implemented in R library hopach [37]. This approach for determining optimal number of clusters can be also used with any other clustering algorithm or distance metric.

### Activity of regulatory clusters

We took the following approach to measure activity of a subnetwork. Let's denote **r** *_i_* - vector of log-ratios of all genes downstream from *i*-th regulator. For each regulator we computed median of log-ratios of downstream genes multiplied by the total number of downstream genes, *K_i_* = *median*(**r** *_i_*)×{**r** *_i_*}. Value *K_i_* reflects contribution of *i*-th regulator into global pattern of differential gene expression. In order to get contribution for entire *j*-th cluster of regulators we summed corresponding *K*-values for all *N_j_* regulators belonging to the cluster, 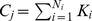. Hence, each sample can be characterized by specifying vector of *C_j_* values for all clusters of regulators.

### Sample clustering (unsupervised analysis)

In order to group patients with similar activity profiles of regulator clusters Ward's clustering was utilized. For comparison with other methods we chose PAM method [36], since it is was found showing consistently good results for microarray data [38] and recently introduced GSVA method [21] with default settings. For both types of cluster analysis Pearson correlation was used as a distance measure.

Adjusted Rand index was used for quantitative comparison of different approaches for patients subtyping: proposed method based on SNEA, traditional methodology based on PAM clustering of gene expression data and GSVA algorithm. In order to assess the reproducibility of clustering we performed 1000 bootstrap runs, each time taking 90% of all available samples from original dataset and calculating Rand index. Thus for each method we obtained distribution of Rand indexes and estimated 95%-confidence intervals for means.

### Feature selection (supervised analysis)

We used Fisher's discriminant criterion to measure the discriminatory power of *i*-th cluster of regulators between classes *a* and *b* having means *μ_i_*_,*a*_, *μ_i_*_,*b*_ and variances 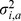, 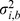, 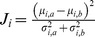. The cluster of regulators was considered as significantly correlated with class if p-value for permutation test (1000 permutations of sample labels) was smaller than 0.05 in more than 90 out of 100 runs of 10-fold sampling without replacement. We also calculated the area under ROC-curve (AUC) for final evaluation of significant features.

### Software

We used Pathway Studio 9.0 from Elsevier [35] to run SNEA. The post-processing of SNEA results was implemented as a set of R scripts (R Development Core Team, www.r-project.org). We also developed simple application that serves as a graphical user interface to scripts, allowing user-friendly specification of algorithm parameters. Scripts generate a number of plots and tables which contain the detailed description of the obtained clustering of regulators and samples. Developed software is intended to work only with Pathway Studio-generated SNEA results and is available at www.sourceforge.net/projects/bsnea.

## Results

**Figure 1:**
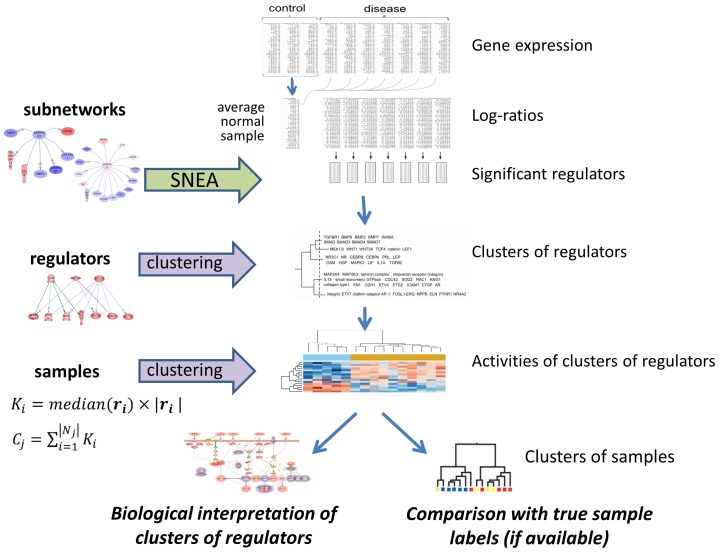
Overall pipeline of the proposed approach for disease subtyping. See corresponding section for detailed description.

The overall pipeline of the study is provided on Figure 1. Analysis starts from expression dataset which should contain control group of samples and samples from patients suffering from a disease. The ultimate goal of the analysis is to find molecular subtypes of studied disease which result in different patterns of changed gene expression and regulation and correlate with clinical outcomes.

Data from control group are averaged to create a standardized control sample. Disease samples are compared with this average control sample and log-ratios for each gene are calculated. Obtained log-ratios are used to run SNEA procedure, which results in the lists of significant regulators. For each disease sample we retain top 100 regulators with p-value smaller than 0.05.

The next step of the approach is to group regulators in clusters. There are several reasons to do this. We found that due to biological variability in different samples SNEA often identifies different regulators although belonging to the same pathway. For example let SMAD3 to be found as a significant regulator in one sample, while in another sample SMAD4 is identified. Both regulators are related to the same pathway - TGFβ signaling - and it would be more reasonable to say that in both samples TGFβ pathway is activated. Thus we propose clustering of regulators having similar sets of downregulated genes. This also makes biological sense, since transcription factors (which constitute the majority of regulators) are known to be redundant - one family member can buffer the loss of another [39]. Clustering of regulators can be viewed as another step in biological-driven feature selection since from thousands of genes we move to hundreds of regulators and further to dozens of clusters of regulators. We also found that overall accuracy of disease subtyping increases when using clusters of regulators instead of regulators themselves.

Regulators identified in all samples are grouped together using Ward's clustering and similarity between two regulators is defined as fraction of common-regulated downstream genes. Optimal number of clusters is estimated using maximum average silhouette method [36].

Each cluster of regulators is assigned a value describing cluster's activity in given sample based on expression of the constituent genes. For each regulator we calculate median expression of downstream genes multiplied by the number of such genes. Then, to define signature for cluster of regulators, we sum corresponding values for each regulator in cluster. Thus initial matrix *N*×*m*, where *N* is number of genes and *m* – number of patients is transformed into matrix *r*×*m*, where *r* is number of clusters of regulators, and *r*<<*N*. Finally obtained matrix of clusters of regulators activities is used to group patients in order to find disease subtypes. Again Ward's clustering is applied and Pearson correlation is used as a similarity measure.

There can be two types of evaluation and interpretation of obtained results. First we can compare correspondence between predicted clusters of samples and true sample labels (if available). For this purpose we utilize Rand index, which gives the measure of coincidence between two partitions. We've used extensive cross-validation of the whole workflow, and performed 100 runs of 10-fold cross-validation calculating Rand index for each run. This makes possible statistical evaluation of the difference between several approaches to disease subtyping by comparing the distributions of Rand indexes.

Secondly we are interested in understanding the biological meaning of clusters of regulators which are significantly deregulated between groups of patients. This includes closer inspection of specific regulators from the discriminating clusters including their genetic alterations and expression changes. We propose that identified clusters of regulators discriminating between groups can be used to discover molecular mechanisms responsible to a specific condition. Below we show the application of the described approach.

### Case study 1: expression analysis of colon biopsies

We used GEO dataset GSE4183 [32] which describes gene expression in colonic biopsies of 15 patients with colorectal carcinoma, 15 with precancerous adenoma, 15 with inflammatory bowel diseases and 8 healthy normal controls. For each gene we calculated the expression variance for controls and for combined disease samples. We found that for 81.3% of all genes the within-group variance was lower than between-group variance, thus providing support for averaging of expression values of genes in healthy samples.

**Figure 2:**
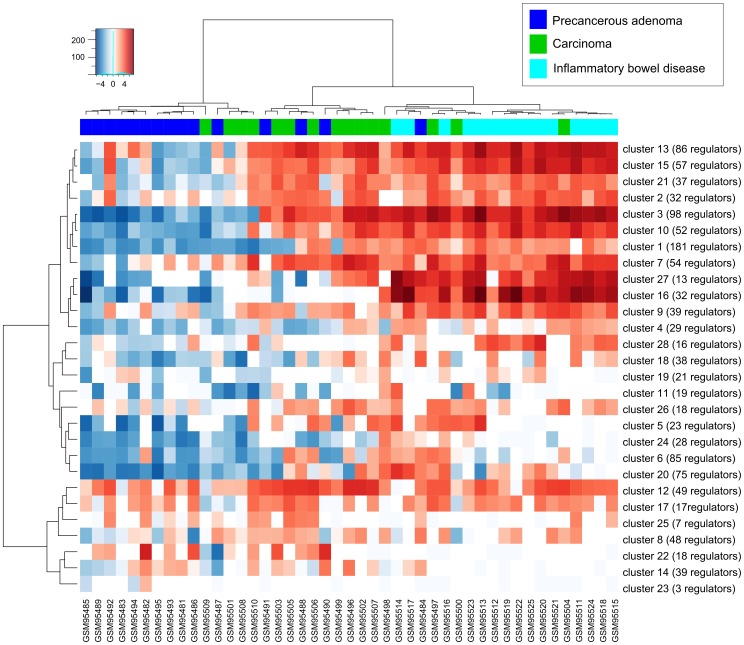
Heatmap of activity scores (k-values) for clusters of regulators identified in GSE4183 dataset. Samples are in columns, clusters of regulators are in rows. Horizontal side bar color encodes true class labels.

SNEA analysis revealed total 1214 expression regulators which were grouped in 28 clusters (Table S1, Figure S1). Activity scores (k-values) from 28 clusters of regulators were used to subdivide samples into 3 groups (see Figure 2).

We found that proposed regulator activity-based patient clustering outperformed both PAM and GSVA method: the average Rand index for 100 runs of 10-fold cross-validation for our method was 0.370±0.014, while the same value for PAM clustering was 0.320±0.009 and for GSVA corresponding mean Rand index was 0.349±0.015.

Next we turned to determination of clusters of regulators which discriminate between conditions: carcinoma, adenoma and inflammatory bowel diseases since this may help find molecular disease-specific mechanisms. Fisher discriminant criterion was utilized to measure the discriminatory power of each cluster of regulators and the significance was determined using permutation test. We also calculated the area under ROC-curve (AUC) for significantly discriminating clusters of regulators.

The resulting table (Table 1) shows that there is one discriminating cluster (#10) of regulators, significant for all pair-wise condition comparisons. Another observation is that in terms of regulation a lot of clusters can discriminate inflammatory disease from both carcinoma and adenoma (#10, #17, #15, #27, #13, #3, #4, #28, #1) or adenoma alone (#7, #9, #24, #21, #11, #6, #2, #5), whereas only two of them (#10 and #7) can help in discrimination of carcinoma vs adenoma. It also can be seen, that carcinoma has more “inflammatory” features (has closer profile to inflammation) than adenoma.

**Table 1:** Identified significant clusters of regulators discriminating between adenoma, carcinoma and inflammation.

Cluster of regulators	Number of regulators in cluster	Inflammation vs adenoma, AUC	Carcinoma vs adenoma, AUC	Inflammation vs carcinoma, AUC
cluster #10	52	0.991	0.742	0.707
cluster #17	32	0.991	–	0.947
cluster #15	57	0.938	–	0.787
cluster #27	13	0.893	–	0.778
cluster #13	86	0.947	–	0.707
cluster #7	54	0.884	0.760	–
cluster #3	82	0.991	–	0.556
cluster #4	29	0.769	–	0.689
cluster #28	16	0.796	–	0.636
cluster #1	181	0.769	–	0.340
cluster #9	39	0.876	–	–
cluster #24	28	0.867	–	–
cluster #21	37	0.813	–	–
cluster #11	45	0.813	–	–
cluster #6	111	0.742	–	–
cluster #2	32	0.662	–	–
cluster #5	23	0.422	–	–

Among the clusters that could help to differentiate carcinoma from adenoma there are clusters #10 and #7. Cluster #10 contains 6 TFs out of 52 regulators: NFATC2 (nuclear factor of activated T-cells, cytoplasmic, calcineurin-dependent 2), FOXP3 (forkhead box P3), RELB (v-rel reticuloendotheliosis viral oncogene homolog B), TBX21 (T-box 21), IRF4 (interferon regulatory factor 4), T (T, brachyury homolog (mouse)), and all of them, along with numerous interleukins and T cell-surface proteins from this cluster are related to the T-cell activation, cytokine production and immune response. Indeed, in the agreement with analyzed data (see Figure 2), T-cell activation is obviously implicated in inflammatory disease [40], as well as in colorectal carcinoma [41]. Moreover, it is shown elsewhere that some of the T-helpers are highly activated throughout the colorectal adenoma-carcinoma sequence [42]. Despite the fact that inflammatory response can be secondary effect of the cancer development, activation of this cluster could be further evaluated as a prognostic factor of the disease progression as well as a potential predictor for the suggested pharmacological intervention in corresponding patients. To support the last statement one could take into account that, for example, antagonists of the NFAT family of transcription factors are known to exhibit strong antineoplastic promoting activity (for review see [43]), and anti-CCR4 mAb selectively depletes effector-type FoxP3+CD4+ regulatory T cells, evoking anti-tumor immune responses in humans [44].

Taking a closer look at the specific regulators from the discriminating cluster may provide a valuable hypothesis about possible sample-specific disease mechanisms and drug targets in the context of the corresponding cell processes. Hence we have searched for frequent known genetic alterations in these regulators using cBioPortal [45]. The most frequently affected regulator from cluster #10 is NFATC2 that was altered by either gene amplification or mRNA upregulation in 33% of reported cases [46]. It was also reported [47] on experimental model of colitis-associated colorectal carcinoma, that NFATC2-deficient mice were protected from tumor development and show significantly reduced levels of the downstream critical proinflammatory cytokines interleukin IL21 and IL6. In studied experiment, GSE4183, NFATC2 was found as significant regulator of downregulated genes in only two cases GSM95508, GSM95509, and for both of them expression of IL6 and IL21 is either downregulated or changed insignificantly compared to the normal samples and both of them are classified as “carcinoma”, that looks to be counter-intuitive. However, looking at unsupervised clustering (Figure 2), one can see that these two samples clearly belong to the “precancerous adenoma” cluster of samples. This is confirmed by results of 1000 bootstrap runs each time taking 90% of all available samples from original dataset - we found that GSM95508 and GSM95509 were classified as belonging to “adenoma cluster” in 70.8% and 98.2% of all runs respectively. We can speculate that more “adenoma-like” and not “carcinoma-like” molecular profile of these samples could be due to the reduced activity of NFATC2. This example may be taken as a use-case for personalized approach to generating hypotheses about activated molecular mechanisms behind the disease progression.

Cluster #7 (differentiating inflammation from adenoma and carcinoma from adenoma) contains 3 TFs out of 54 regulators: EPAS1 (endothelial PAS domain protein 1), ETV4 (ets variant 4), CITED2 (Cbp/p300-interacting transactivator, with Glu/Asp-rich carboxy-terminal domain, 2), and different factors, ECM and membrane proteins, like CYR61, matrix metallopeptidases, different PDGFs, PDGFRs, etc. The processes related to the activity of these regulators are vascularization, cell survival and cell migration. One can hypothesize here that the activation of this cluster is associated with angiogenesis and in the case of carcinoma with metastasis and invasion. This observation is in agreement with the paper [48] where authors have shown that the expression of the "angiogenesis" gene set is significantly increased in CRCs compared to adenomas and that the increased mRNA expression levels of PDGFRB (changed in 8.7% of adenocarcinoma cases [46] according to cBioPortal) can be used as a tumor biomarker. Among the other regulators from cluster #7 found as frequently changed according to cBioPortal, there is, for example, frequently mutated or upregulated TNC (changed in 12.8% of adenocarcinoma cases), tenascin C, that promotes the expression of matrix metallopeptidases and was proposed as prognostic biomarker of CRC in many studies [49], [50]. Another example is transcriptional factor, responsible for the activation of VEGF and angiogenesis, EPAS1 (hypoxia inducible factor 2a, changed in 8.2% of cases), that is once expressed in stroma is associated with a poorer prognosis in colorectal cancer [51].

Overall, both clusters, taken as an example, correspond to the known to be significant cancer-related processes, and the regulators, found within these clusters are frequently proposed as colorectal cancer biomarkers. Moreover, taking into account genetic alterations of these regulators, we speculate that it may be possible to propose the single-patient hypothesis, based on the combination of his mutational status and the expression patterns.

### Case study 2: clustering of 12 human neuromuscular diseases

We also demonstrate proposed approach for clustering of regulators using a larger number of diseases. Here the task is to group together diseases with similar patterns of expression regulation rather than patients. In this case we cannot make use of Rand index to assess the overall performance, since the correct answer is not known. Instead we evaluate resulting disease clusters based on biological expertise and compare our approach with traditional expression-based clustering.

We used GEO dataset GSE3307 [33] which contains comparative profiling of total 121 samples of human skeletal muscle in 13 patient groups. Known diagnostic groups included 18 samples of normal human skeletal muscle, 5 patients with acute quadriplegic myopathy (AQM), 21 patients with juvenile dermatomyositis (JDM), 9 patients with amyotophic lateral sclerosis (ALS), 4 patients with hereditary spastic paraplegia (HSP), 8 patients with Emery-Dreifuss muscular dystrophy (EDMD, 4 samples of X linked recessive emerin form and 4 samples of autosomal dominant lamin A/C form), 7 patients with dystroglycanopathy caused by mutation in FKRP protein, 5 patients with Becker muscular dystrophy (BMD), 10 patients with Duchenne muscular dystrophy (DMD), 10 patients with calpain 3 deficiency, 10 patients with dysferlin 3 deficiency and 14 patients with fascioscapulohumeral muscular dystrophy.

We calculated mean expression for each gene within every group to obtain “average” patient representing the whole disease and run the pipeline on these data. Thus we compared neuromuscular diseases between each other rather than patients. Total 714 expression regulators were identified which were subsequently grouped in 34 clusters. We performed Ward's clustering for 12×34 matrix of activity scores for clusters of regulators (k-values). For the reference the same type of cluster analysis was performed for data matrix of gene expression values. In both cases Pearson correlation was used as a distance measure.

**Figure 3:**
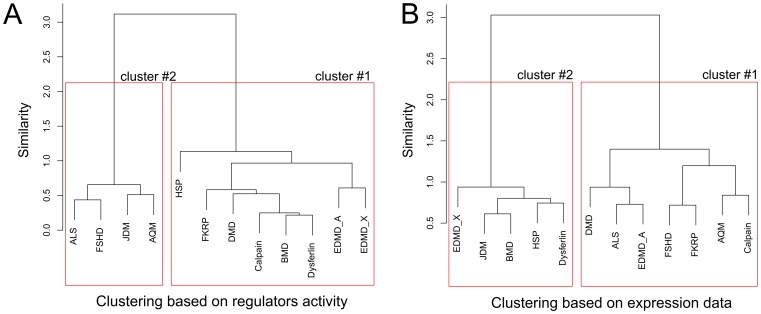
Comparison of clustering of 12 diseases of human muscle. A) Dendrogram obtained using proposed approach based on analysis of regulators activity. B) Dendrogram obtained using Ward's method for clustering gene expression data.

Obtained dendrograms for both approaches to finding similar diseases are shown on Figure 3. Overall results of our analysis (Figure 3, A) suggest that human muscle-related pathologies can be clustered in two large groups. Cluster №1 includes mostly dystrophic myopathies, inherited single gene disorders causing degeneration of muscle fibers: DMD, BMD, FKRP, calpain 3 and dysferlin deficiencies. It can be viewed as cluster of diseases which are triggered by structural damage caused by mutation in a single gene. Cluster №2 includes non-muscular diseases (amyotrophic lateral sclerosis, death of motor neurons mainly due to the unknown reasons but with some familial (i.e. SOD1 or C9orf72-mutant) cases [52]; juvenile dermatomyositis, an autoimmune disease of unknown cause [53]) or diseases with primarily unknown mechanisms that may be related to functional disorders in the organism or complex genetic events (acute quadriplegic myopathy, preferential loss of myosin and myosin-associated thick filament proteins [54]; fascioscapulohumeral muscular dystrophy - progressive weakness of the upper body muscles due to deletions of the D4Z4 repeat located in the terminal region of chromosome 4 [55]).

Furthermore, close inspection of cluster's fine structure also reveals biologically meaningful patterns. For example two nuclear envelope defects caused by mutations in LMNA and emerin gene are close to each other on the dendrogram and hence are predicted to be disorders having much in common in terms of activated signaling cascades and molecular mechanisms. Indeed both mutations lead to Emery–Dreifuss muscular dystrophy phenotype. This is supported by results previously reported in [33] where the authors took completely different approach for gene expression analysis based on decision trees. Another prediction suggests interconnectivity between two diseases caused by calpain 3 and dysferlin deficiencies. Indeed, mutations in both genes lead to limb-girdle muscular dystrophy, 2A and 2B respectively. It was shown by co-immunoprecipitation experiments that calpain 3 is in complex with dysferlin [56]. However some predictions are hard to interpret, for example, the similarity of hereditary spastic paraplegia caused by mutation in SPG4 gene and dystroglycanopathy caused by mutation in FKRP protein, involved in glycosylation of dystroglycan.

On the contrary, inspection of cluster composition obtained using expression data (Figure 3b) shows that these results are much less biologically reasonable. For example pathogenetically similar DMD and BMD (which both result from a mutation in the *dystrophin* gene) are in different clusters. The same is true for different forms of Emery–Dreifuss muscular dystrophy (EDMD-X and EDMD-A).

Finally we conclude that proposed approach gives biologically meaningful grouping of diseases and in this sense outperforms traditional method based on gene expression clustering.

## Discussion

In this paper we have proposed novel approach to disease subtyping based on Subnetwork Enrichment Analysis. We use pathway activity inferred in each sample separately to cluster patients together thus performing clinical classification. We emphasize that our method is not related to establishing pathways from expression data [57]. Instead expression of thousands of genes is used to infer activity of much fewer significant regulators using global network of literature-extracted protein regulation relations.

Analysis at the level of expression regulators facilitates interpretation of transcriptomics data giving biological explanation to observed changes in expression of hundreds of genes. Although regulators themself are rarely differentially expressed they are the driving force behind real molecular processes in the cells. In many cases mutations in regulators are the key reasons for the development of the diseases (an obvious example is the connection between mutations in *p53* and cancer progression). Also regulators being the hubs in protein-protein interaction networks often serve as a drug targets. Thus inferring regulators from transcriptomics data gives another layer of biological information complementary to gene expression.

Finally we'd like to summarize distinctive features of the proposed method for disease subtyping which combines advantages of gene set enrichment methods with information about topology of global literature-extracted protein regulation network.

At first, there's no need for a priori defined functional gene sets or pathway collections which are almost inevitably biased to more studied diseases/conditions. Using SNEA for gene expression data allows quick identification of the regulators and exploratory biomarkers [58]. Deregulated subnetworks consisting of regulator and downstream genes are identified for each patient separately. This provides basis for personalized treatment since each regulator may be a marker of activated molecular mechanism behind a disease progression in individual.

At the same time clustering of regulators identified in all samples reduces effects of variability and noise in the data. Obtained clusters of regulators suggest rational biological interpretation of observed changes in gene expression. In addition pool of possible regulators is not limited to transcription factors and can include entities (functional classes, complexes, etc) which are not measured directly in the experiment. Clusters of regulators affecting mainly the same set of genes can be interpreted as a first approximation to bottom-up automatic reconstruction of pathways.

Algorithm performance heavily depends on Resnet [30], [59], [60], [61], global literature-derived network of over 1,500,000 relations which were extracted by automatic analysis of more than 22 million PubMed abstracts and 880,000 full-text articles. Many relations between entities stored in Resnet database are indirect (for example, expression links between non-transcription factors and downstream genes) and can be obtained only from literature-based analysis and not from experimental data.

A distinctive feature of our approach is that it utilizes cluster analysis, being an unsupervised technique. While the method needs information whether each sample belongs to “normal” or “disease” group, there's no need to specify different subgroups within a “disease” class or use a training set. We believe that the main field of use for proposed approach is to predict and characterize phenotypes in clinical outcome studies: predict responders and non-responders to specific treatment, survival time, perform a differential diagnosis, etc. For example we applied our approach for identification of molecular mechanisms activated in nonresponders to cetuximab treatment (data not shown).

We note that proposed method is not limited to microarray as an experimental technique to quantify gene expression. The whole pipeline can be run using RPKM values from RNASeq data as input. However to our knowledge there are no publicly available RNASeq datasets that satisfy our criteria: more than two classes and relatively large number of samples with clinical annotations. Application of our approach to this type of data remains area for future research.

In concluding, we propose novel unsupervised method for disease subtyping based on analysis of activated gene expression regulators on a sample-by-sample basis. We show that our approach for patient stratification performs well compared with traditional expression-based clustering. However the main benefit of method is that identified clusters of expression regulators provide valuable insight into pathway-level understanding of biology related to a clinical outcome for individual patient.

## Supporting Information

Figure S1 **Clustering of regulators for GSE4183 dataset.** Dendrogram obtained using Ward's clustering and Jaccard distance. Clusters are shown in red.
(PDF)

Table S1 **Identified clusters of regulators for GSE4183 dataset.** Column description: **Regulator** – name of identified regulator (from Resnet database). **Cluster** # – number of cluster to which regulator is assigned. **Is transcription factor** – whether identified regulator is a transcription factor.
(XLSX)
