# Supplementary material for: Clustering Gene Expression Regulators: New Approach to Disease Subtyping
Source: PLoS One. 2014 Jan 9;9(1):e84955. doi: 10.1371/journal.pone.0084955 (PMC3887006; doi:10.1371/journal.pone.0084955)

Regulators similarity(1214), dist=Jaccard, clust=ward

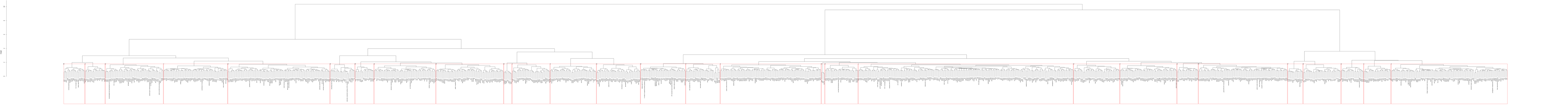

Supplement: Figure S1 — Clustering of regulators for GSE4183 dataset. Dendrogram obtained using Ward's clustering and Jaccard distance. Clusters are shown in red. (PDF) [file pone.0084955.s001.pdf]
